# Induction of proto-oncogene BRF2 in breast cancer cells by the dietary soybean isoflavone daidzein

**DOI:** 10.1186/s12885-015-1914-5

**Published:** 2015-11-16

**Authors:** Jana Koo, Stephanie Cabarcas-Petroski, John L. Petrie, Nicole Diette, Robert J. White, Laura Schramm

**Affiliations:** Department of Biological Sciences, St. John’s University, Queens New York, 11439 USA; Pennsylvania State University, Beaver Campus, Monaca, PA 15061 USA; Department of Biology, University of York, Heslington, York, YO10 5DD UK

**Keywords:** Breast Cancer, TFIIIB, BRF2, RNA polymerase III, Soy, Daidzein

## Abstract

**Background:**

BRF2 is a transcription factor required for synthesis of a small group of non-coding RNAs by RNA polymerase III. Overexpression of BRF2 can transform human mammary epithelial cells. In both breast and lung cancers, the BRF2 gene is amplified and overexpressed and may serve as an oncogenic driver. Furthermore, elevated BRF2 can be independently prognostic of unfavorable survival. Dietary soy isoflavones increase metastasis to lungs in a model of breast cancer and a recent study reported significantly increased cell proliferation in breast cancer patients who used soy supplementation. The soy isoflavone daidzein is a major food-derived phytoestrogen that is structurally similar to estrogen. The putative estrogenic effect of soy raises concern that high consumption of soy foods by breast cancer patients may increase tumor growth.

**Methods:**

Expression of BRF2 RNA and protein was assayed in ER-positive or –negative human breast cancer cells after exposure to daidzein. We also measured mRNA stability, promoter methylation and response to the demethylating agent 5-azacytidine. In addition, expression was compared between mice fed diets enriched or deprived of isoflavones.

**Results:**

We demonstrate that the soy isoflavone daidzein specifically stimulates expression of BRF2 in ER-positive breast cancer cells, as well as the related factor BRF1. Induction is accompanied by increased levels of non-coding RNAs that are regulated by BRF2 and BRF1. Daidzein treatment stabilizes BRF2 and BRF1 mRNAs and selectively decreases methylation of the BRF2 promoter. Functional significance of demethylation is supported by induction of BRF2 by the methyltransferase inhibitor 5-azacytidine. None of these effects are observed in an ER-negative breast cancer line, when tested in parallel with ER-positive breast cancer cells. *In vivo* relevance is suggested by the significantly elevated levels of BRF2 mRNA detected in female mice fed a high-isoflavone commercial diet. In striking contrast, BRF2 and BRF1 mRNA levels are suppressed in matched male mice fed the same isoflavone-enriched diet.

**Conclusions:**

The BRF2 gene that is implicated in cancer can be induced in human breast cancer cells by the isoflavone daidzein, through promoter demethylation and/or mRNA stabilization. Dietary isoflavones may also induce BRF2 in female mice, whereas the converse occurs in males.

## Background

RNA polymerase (pol) III has the responsibility of synthesizing a variety of short noncoding RNAs such as tRNAs and the spliceosomal U6 snRNA [1]. Initiation by pol III requires TFIIIB [1], a transcription factor complex with at least two forms in mammalian cells [2, 3]. Gene-internal pol III promoters, such as those found in tRNA genes, require TFIIIB composed of TBP, BDP1 and BRF1 subunits, whereas gene-external pol III promoters, as exemplified by U6 genes, require TFIIIB containing TBP, BDP1 and BRF2 [1]. Aberrant pol III transcription is a feature of many tumor types [4]. This reflects, in part, the fact that TFIIIB is strongly regulated by pathways involving oncogenes and tumor suppressors [4, 5]. For example, MYC [6] and the MAP kinase ERK [7] bind to TFIIIB and stimulate its activity, whereas an array of tumor suppressors inhibit TFIIIB activity [8], either directly or indirectly, including BRCA1 [9], PTEN [10, 11], p53 [12], and the RB family [13].

The BRF2 subunit of TFIIIB is encoded by an oncogene at 8p12 that is frequently amplified and overexpressed in breast cancers and lung squamous cell carcinomas (SqCC) [14–20, 21]. BRF2 drives the 8p12 amplification in SqCC [22]. Its overexpression stimulates proliferation and saturation density of human bronchial epithelial cells, whereas its knockdown specifically suppresses proliferation and anchorage-independent growth of SqCC cells with 8p12 amplification [22]. Copy number increases and overexpression of BRF2 are apparent in most pre-invasive bronchial carcinomas *in situ*, with minimal staining in benign lesions [22]. *BRF2* induction was therefore proposed as an early event in development of lung SqCC, that might serve as a marker and/or therapeutic target [22]. Subsequent independent studies reported elevated BRF2 protein in lung and esophageal SqCC, where high BRF2 was independently prognostic of unfavorable survival for both lung (*P* = 0.007) and esophageal (*P* = 0.009) SqCC [23, 24]. BRF2 overexpression may also be an oncogenic driver in some breast cancers and human mammary epithelial cells can be transformed by transfection of the BRF2 gene [15]. Analysis of published datasets, using the Web-based Oncomine platform, reveals that BRF2 was amongst the top 1 % of genes overexpressed in a study [25] of 154 invasive breast carcinomas (*p* = 3.53E-10), whilst a larger study of over two thousand breast samples [26] confirmed BRF2 overexpression in several tumor subgroups, with invasive ductal breast carcinomas the most significant (*p* = 2.17E-21). The cBioPortal cancer genomics database [27–29] reveals amplification of the BRF2 gene in 12 % of 825 tumors in the Breast Invasive Carcinoma study (TCGA, Nature 2012) [30].

The chemopreventive polyphenol EGCG, enriched in green tea, specifically decreases TFIIIB activity in cervical cancer cells [31]. The polyphenols genistein and daidzein are isoflavone components of soybeans, a major crop in the United States and globally [32]. These soy isoflavones are major food-derived phytoestrogens that are structurally similar to estrogen with the capacity to weakly bind to estrogen receptors (ERs) [33]. The putative estrogenic effect of soy raises the concern that high consumption of soy foods by breast cancer patients and/or women at high risk for breast cancer may increase estrogen-dependent breast tumor growth [34]. A recent study reported a significant increase in cell proliferation in breast cancer patients who used soy supplementation [35]. Dietary soy isoflavones increase metastasis to lungs in an experimental model of breast cancer [36]. These data prompted us to investigate if the soy isoflavone daidzein regulates TFIIIB. We found that 10 uM daidzein stimulates expression of the TFIIIB subunits BRF1 and BRF2 in ER-positive breast cancer cells, as well as pol III products U6 snRNA and tRNA_i_^Met^. Daidzein treatment stabilizes BRF2 and BRF1 mRNAs and raises levels of their protein products. It also triggers selective demethylation of the BRF2 promoter. These effects are not seen in an ER-negative breast cancer line. An isoflavone-enriched diet also induces BRF2 in female mice, but has the opposite effect in males. These *in vitro* and *in vivo* data suggest that dietary isoflavones differentially regulate TFIIIB expression, an important observation given the evidence that BRF2 can drive tumorigenesis and is predictive of poor prognosis.

## Methods

### Cell lines and daidzein treatment

MCF-7 and MDA-MB-231 cells were obtained from the American Type Culture Collection (Rockville, MD). Cells were cultured in DMEM supplemented with FBS (5 % v/v), nonessential amino acids (100 mM), L-glutamine (5 mM), streptomycin (100 μg/ml), and penicillin (100 units/ml); all from BioWhittaker, Walkersville, MD. Cells were grown at 37 °C in a humidified atmosphere of 95 % air and 5 % CO_2_ as previously described [37, 38]. Daidzein (Sigma) treatments are as described in figure legends.

### 5-Azacytidine treatment

Asynchronous MCF-7 and MDA-MB-231 cells were plated at 1 × 10^4^ cell/well in 6-well plates. After 24 h, cells were treated with 5 μM 5-azacytidine (Sigma) for 24, 48 and 72 h. At each time point, total RNA was collected using RNeasy total RNA isolation kit (Qiagen), according to the manufacturer's protocol and cDNA subsequently prepared to be used in qRT-PCRassays.

### Quantitative reverse transcription PCR (qRT-PCR)

Total RNA was extracted from cancer cell lines using the RNeasy total RNA isolation kit (Qiagen), according to the manufacturer's protocol and qPCR was performed using diluted cDNA from treated breast cancer cells and SsoAdvanced™ Universal SYBR® Green Supermix (BioRad). Gene specific primers include: BRF2-forward, 5’-CAG AAG TGG AGA CCC GAG AG-3’; BRF2-reverse, 5’-CAG GGA GGG TTA GGG ACA CT-3’; BRF1-forward, 5’-GGC ATT GAT GAC CTG GAG AT-3’; BRF1-reverse, 5’-ACC AGA GGC CTC AAC CTT TT-3’; BDP1-forward, 5’-TGG AAG AAG CTG GAA GGA GA-3’; BDP1-reverse, 5’-TTC CTC AAT GGC ATC AAT CA-3’; TBP-forward, 5’-CGG CTG TTT AAC TTC GCT TC-3’; TBP reverse, 5’-CTG TTG TTG TTG CTG CTG CT-3’; U6-forward, 5’-GGT CGG GCA GGA AAG AGG GC-3’; U6-reverse, 5’- GCTAAT CTT CTC TGT ATC GTT CC-3’; tRNA_i_^Met^-forward, 5’- CTG GGC CCA TAA CCC AGA G-3’; tRNA_i_^Met^-reverse, 5’-TGG TAG CAG AGG ATG GTT TC-3’; GAPDH-forward, 5’- TCCACCACCCTGTTGCTGTA-3’; GAPDH-reverse, 5’- ACC ACA GTC CAT GCC ATC AC-3’; RPS13-forward, 5’-GTT GCT GTT CGA AAG CAT CTT G-3’; RPS13-reverse, 5’-AAT ATC GAG CCA AAC GGT GAA-3’; actin β-forward, 5’-TAG CGG GGT TCA CCC ACA CTG TGC CCC A-3’; actin β-reverse, 5’- CTA GAA GCA TTT GCG GTG GAC CGA TGG A-3’. Real time quantitative PCR reactions were carried out using the Bio-Rad CFX Connect System. The ΔΔCt method was employed for each gene tested as noted in figures using GAPDH and RPS13 expression levels for normalization. Meta-analysis of data using one-way ANOVA with a Tukey post-test with a 95 % confidence interval (GraphpadPrism3.03, San Diego California USA); * = *p* <0.05; ** = *p* < 0.01; *** = *p* < 0.001.

### Western blot analysis

Total cellular protein was extracted using Cytobuster Protein Extraction Reagent (Merck Millipore; 71009). Proteins were fractionated by SDS-PAGE and transferred to nitrocellulose membranes, which were incubated overnight with antibodies against BRF1 (Bethyl Laboratories; A301-228A), BRF2 and actin (Santa Cruz Biotechnology; sc-390312 and sc-1615). Membranes were then incubated with HRP-conjugated anti-goat (Dako; P0449) anti-rabbit and anti-mouse (Cell Signaling; 7074 and 7076) IgG for 1 h. Bands were visualized using the enhanced chemiluminescence method.

### Methylation analysis

Promoters were analyzed using MethPrimer [39] to identify potential CpG islands. NEBcutter V2.0 [40] was used to identify methylation sensitive restriction enzyme (MSRE) sites sensitive within promoter sequences. Genomic DNA was isolated from MCF-7 and MD-MB-231 cancer cells using DNeasy Blood & Tissue Kit (Qiagen) using the manufacturer’s protocol. Restriction enzymes AciI, AscI, BanI, BfuAI, BsrFI, BsrBI, BseYI, BfuAI, BspEI, Cac81, FspI, NciI, NruI (New England Biolabs) and HpyCH4III/Taal (Fermentas) were used. Restriction digestions were then analyzed by real time PCR using Universal SYBR Green (BioRad) and primers spanning the promoters regions and primers spanning the promoters regions (BRF2-forward, 5’-GGC CTC CAA AAG CGT T-3’; BRF2-reverse, 5’-AGC TGG CTC TGC GAA TAG T-3’; BRF1-forward, 5’-GGG GTT GGG TCC CAG GTC GC-3’; BRF1-reverse, 5’-GTC CTC CAG CAC TGA GCC GC-3’; U6-forward, 5’- AAG TAT TTC GAT TTC TTG GC-3’; U6-reverse, 5’- AAT ATG GAA CGC TTC ACG-3’; tRNA_i_^Met^-forward, 5’-TAG ATA GCA GAG TGG CGC A-3’; tRNA_i_^Met^-reverse, 5’-AAC TCC GAT AGC AGA GGA TG-3’). Results were quantified using the ΔΔCt method and normalized to RPS13 expression levels. Data presented are average of three independent experiments. Statistical analysis was performed using one-way ANOVA with a Tukey’s post-test with a 95 % confidence interval (Graphpad Prism 3.03); * = *p* <0.05; ** = *p* < 0.01; *** = *p* < 0.001.

### mRNA stability of the BRF1 and BRF2 genes

The mRNA stability of BRF1 and BRF2 mRNA was determined following actinomycin D treatment. Actinomycin D was added to a final concentration of 5 μg/ml to block transcription [41]. At 0, 4, 8, 12 and 24 h post-actinomycin D treatment, the cells were harvested, and mRNA was quantified by qRT-PCRas described above. Data show relative expression values at time points indicated with control untreated sample set to 1 at 0 h post-actinomycin D treatment.

### ELISA

BRF2 protein levels were measured from untreated and daidzein treated MCF-7 and MD-MB-231 cells using a human BRF2 ELISA kit (MyBioSource) as per the manufacturer’s protocol. Data presented are average of four independent experiments. Statistical analysis was performed using one-way ANOVA with a Tukey’s post-test with a 95 % confidence interval (Graphpad Prism 3.03); * = *p* <0.05; ** = *p* < 0.01; *** = *p* < 0.001.

### Animals and isoflavone treatment

Female and male C57BL/6 J mice were purchased from Taconic Farms (Germantown, New York). Mice were housed 4 per cage in a temperature-controlled St. John’s University Animal Care Facility with alternating 12:12 h light–dark cycles, with *ad libitum* access to water and commercial Purina 5001 or 5 K96 chows (Fig. 7), as approved in its entirety by SJU IACUC (SJU Protocol number 1831.0). Mice were numbered with permanent marker identification, and acclimated to animal penthouse for 72 h prior to start of experiment. Mice were treated for a total of three weeks and monitored daily for signs of stress. Weekly tail bleeds and a terminal cardiac puncture bleed were collected and analyzed for TFIIIB levels via qRT-PCR.

## Results

### Daidzein induces BRF1, BRF2 and pol III transcript expression in MCF-7 breast cancer cells

**Fig. 1:**
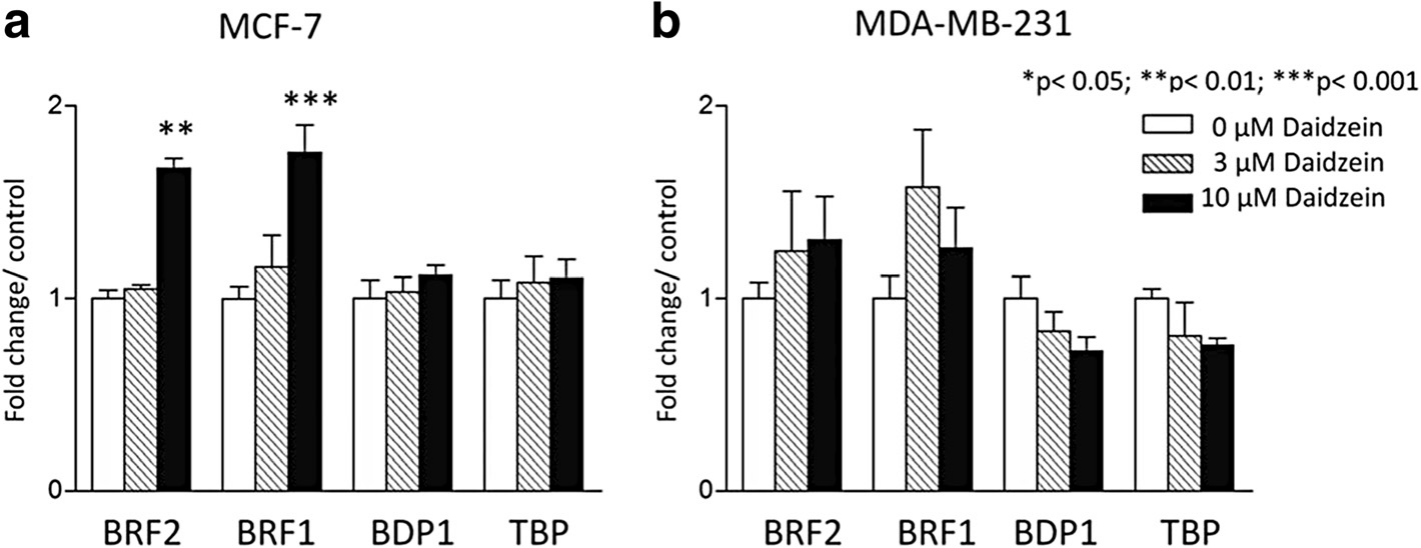
Daidzein induces BRF1 and BRF2 mRNA specifically in MCF-7 breast cancer cells. MCF-7 (**a**) and MDA-MB-231 (**b**) cells were treated with 0, 3 or 10 μM daidzein for 48 h. BRF2, BRF1, BDP1 and TBP mRNA expression was then analysed by qRT-PCRusing the ΔΔCt method with RPS13 expression levels as a reference for normalization. Meta-analysis of three independent experiments performed in triplicate was completed using one-way ANOVA with a Tukey’s post-test with a 95 % confidence interval (Graphpad Prism 3.03); * = *p* <0.05; ** = *p* < 0.01; *** = *p* < 0.001

**Fig. 2:**
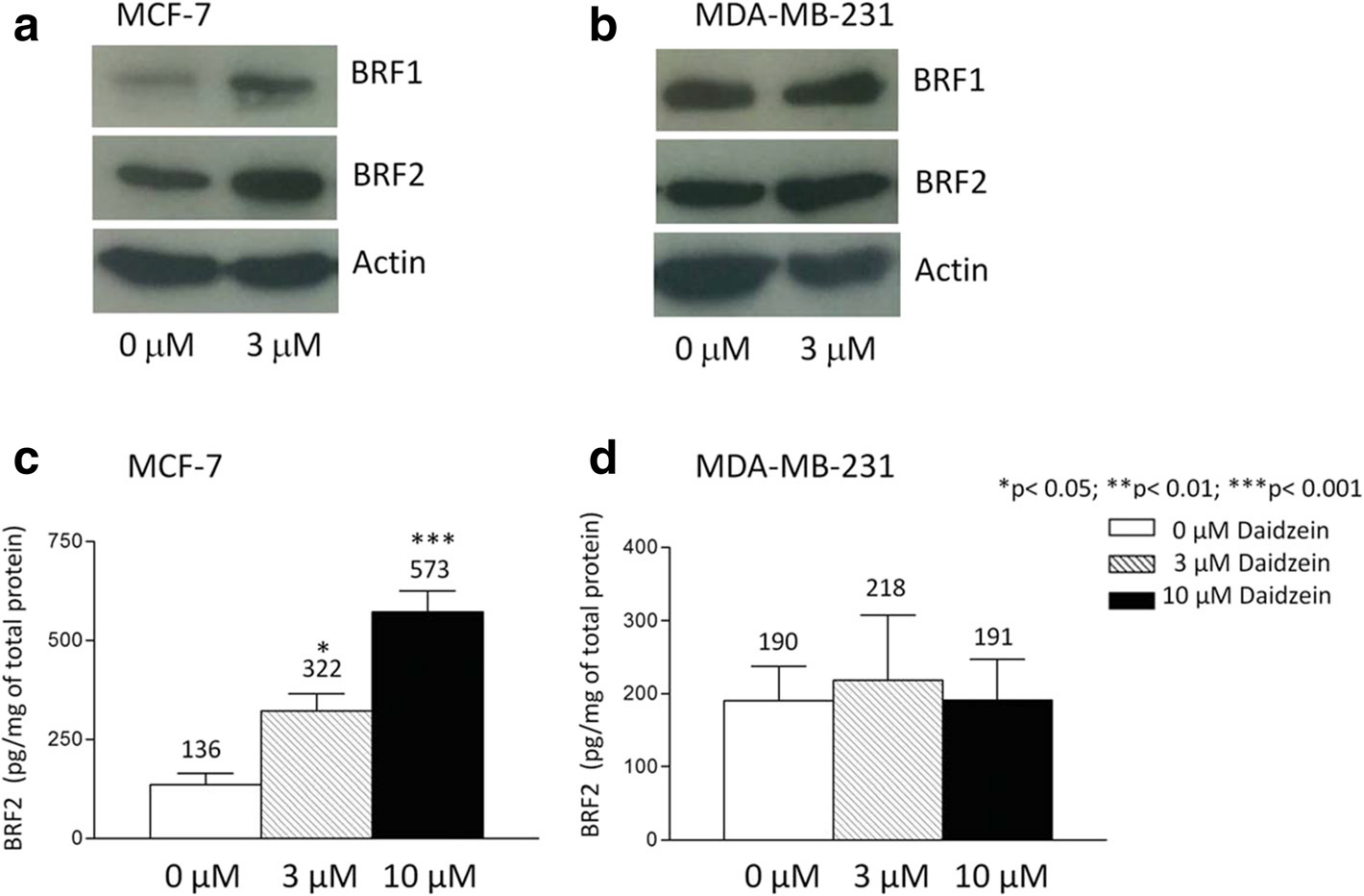
Daidzein induces BRF1 and BRF2 protein specifically in MCF-7 cells. MCF-7 (**a**) and MDA-MB-231 (**b**) cells were treated with 0 or 3 μM daidzein for 48 h and then analysed for expression of BRF1, BRF2 and actin proteins by western blot. Quantification of BRF2 protein expression in (**c**) MCF-7 and (**d**) MDA-MB-231 cells treated with daidzein was achieved by ELISA. Data presented are average of four independent experiments. Statistical analysis was performed using one-way ANOVA with a Tukey’s post-test with a 95 % confidence interval (Graphpad Prism 3.03); * = *p* <0.05; ** = *p* < 0.01; *** = *p* < 0.001

Human breast cancer cell lines were treated for 48 h with daidzein and qRT-PCRwas used to assay expression of mRNAs encoding the subunits of TFIIIB. The mRNAs encoding BRF1 and BRF2 were both found to be induced significantly in ER-positive MCF-7 cells (*p* < 0.001 and *p* < 0.01, respectively; Fig. 1a). This response is selective and not shown by the mRNAs encoding TBP and BDP1, the other TFIIIB subunits. Furthermore, none of the TFIIIB mRNAs showed significant responses to daidzein in ER-negative MDA-MB-231 cells, under these conditions (Fig. 1b). The induction of BRF1 and BRF2 mRNAs in MCF-7 cells is translated into a corresponding increase in the protein products, as shown by western blotting (Fig. 2a). As with the mRNA, protein expression does not respond in MDA-MB-231 cells (Fig. 2b). Quantitative analysis by ELISA established that BRF2 protein induction in MCF-7 cells is 2.4-fold with 3 μM daidzein and 4.2-fold with 10 μM daidzein (Fig. 2c). As observed by western, no significant change in BRF2 levels was detected by ELISA with MDA-MB-231 cells (Fig. 2d). The uninduced level of BRF2 is higher in MDA-MB-231 than MCF-7 cells (1.4-fold elevated by quantitative ELISA), consistent with a previously published report [42], which showed that BRF2 mRNA is expressed at higher levels in MDA-MB-231 cells than in MCF-7 cells. However, it is unlikely that BRF2 has reached a saturation point in untreated MDA-MB-231 cells that would preclude further induction.

**Fig. 3:**
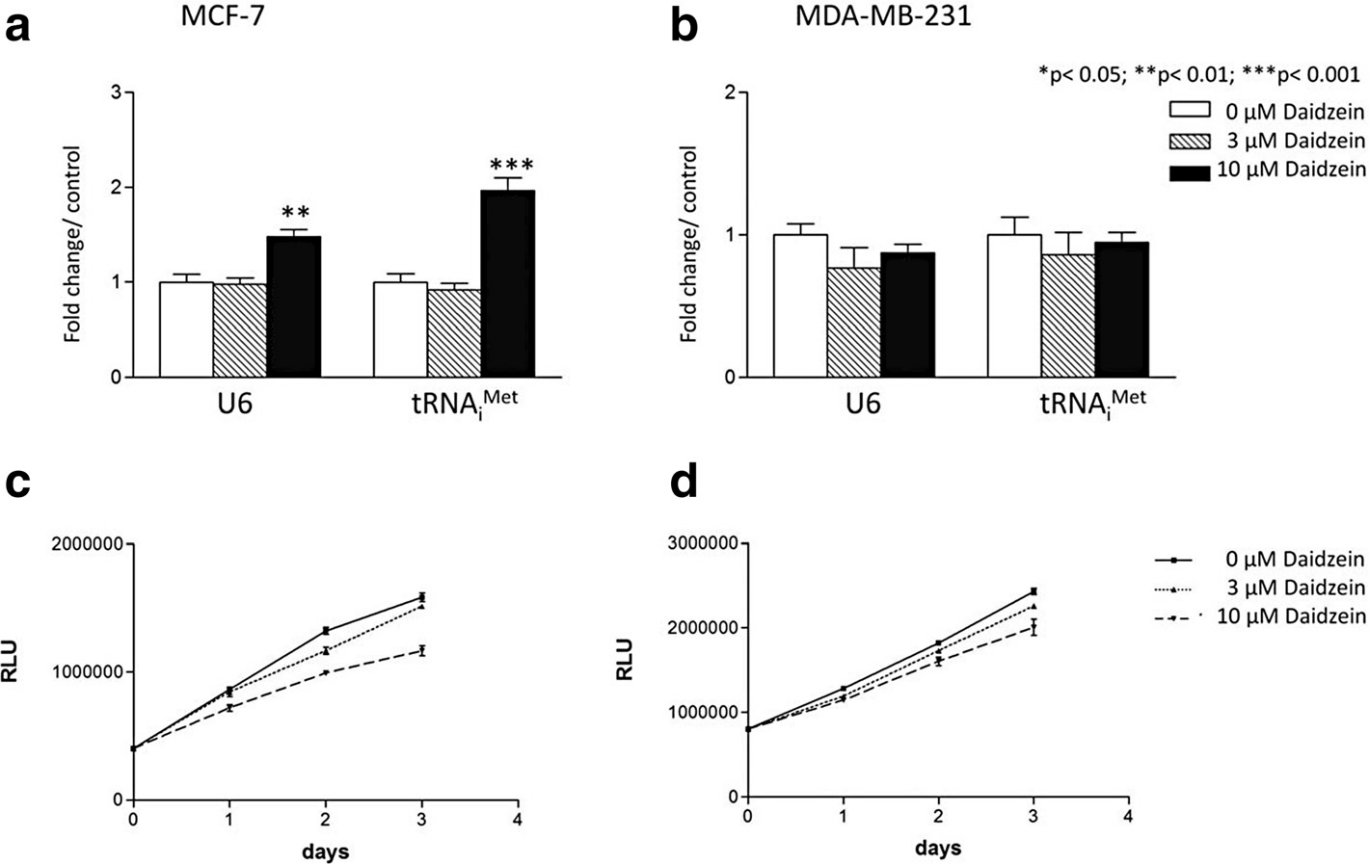
Daidzein induces U6 snRNA and tRNA_i_^Met^ specifically in MCF-7 cells without inducing proliferation. MCF-7 (**a**) and MDA-MB-231 (**b**) cells were treated with 0, 3, 10 μM daidzein for 48 h. U6 snRNA and tRNA_i_^Met^ expression was then analysed by qRT-PCRusing the ΔΔCt method with RPS13 expression levels as a reference for normalization. Meta-analysis of three independent experiments performed in triplicate was completed using one-way ANOVA with a Tukey’s post-test with a 95 % confidence interval (Graphpad Prism 3.03); * = p <0.05; ** = p < 0.01; *** = p < 0.001. CellTiter-Glo® (Promega) was used to count MCF- 7 (**c**) and MDA-MB-231 cells (**d**) after 24, 48 and 72 h treatment with 0, 3 or 10 μM daidzein, as indicated. Each dose and time point was performed in triplicate. MDA-MD-231 cell proliferation significantly decreased with 10 μM daidzein after 48 h (*p* < 0.05) and 72 h (*p* < 0.01) treatment. MCF-7 cell proliferation significantly decreased at 48 h (*p* < 0.01) with 3 μM daidzein treatment. At 10 μM daidzein treatment cell proliferation was significantly inhibited in MCF-7 cells at 24 h (p < 0.05), 48 h (*p* < 0.01) and 72 h (*p* < 0.01)

If the observed induction of BRF1 and BRF2 is functionally significant, we would expect to see changes in expression of pol III products that depend upon these subunits for their transcription. Indeed, both U6 snRNA, dependent on BRF2, and tRNA_i_^Met^, requiring BRF1, show significant induction in MCF-7 cells by 10 μM daidzein (Fig. 3a). As with BRF1 and BRF2, neither of these pol III transcripts is induced when MDA-MB-231 cells are treated in the same way (Fig. 3b). As pol III activity is generally coupled to cell proliferation, we tested if daidzein has a mitogenic effect under our assay conditions. However, cell viability assays provided no evidence of enhanced proliferation when either cell line was exposed to 10 μM daidzein (Figs. 3c and d); indeed, significant suppression was observed after 72 h, in agreement with previous studies noting daidzein treatments greater than 1 μM inhibit breast cancer cell proliferation [43]. The selective induction of U6 snRNA and tRNA_i_^Met^ therefore appears not to reflect a mitogenic response, but correlates with increases in BRF1 and BRF2.

### Daidzein decreases methylation of the BRF2 promoter specifically in MCF-7 cells

**Fig. 4:**
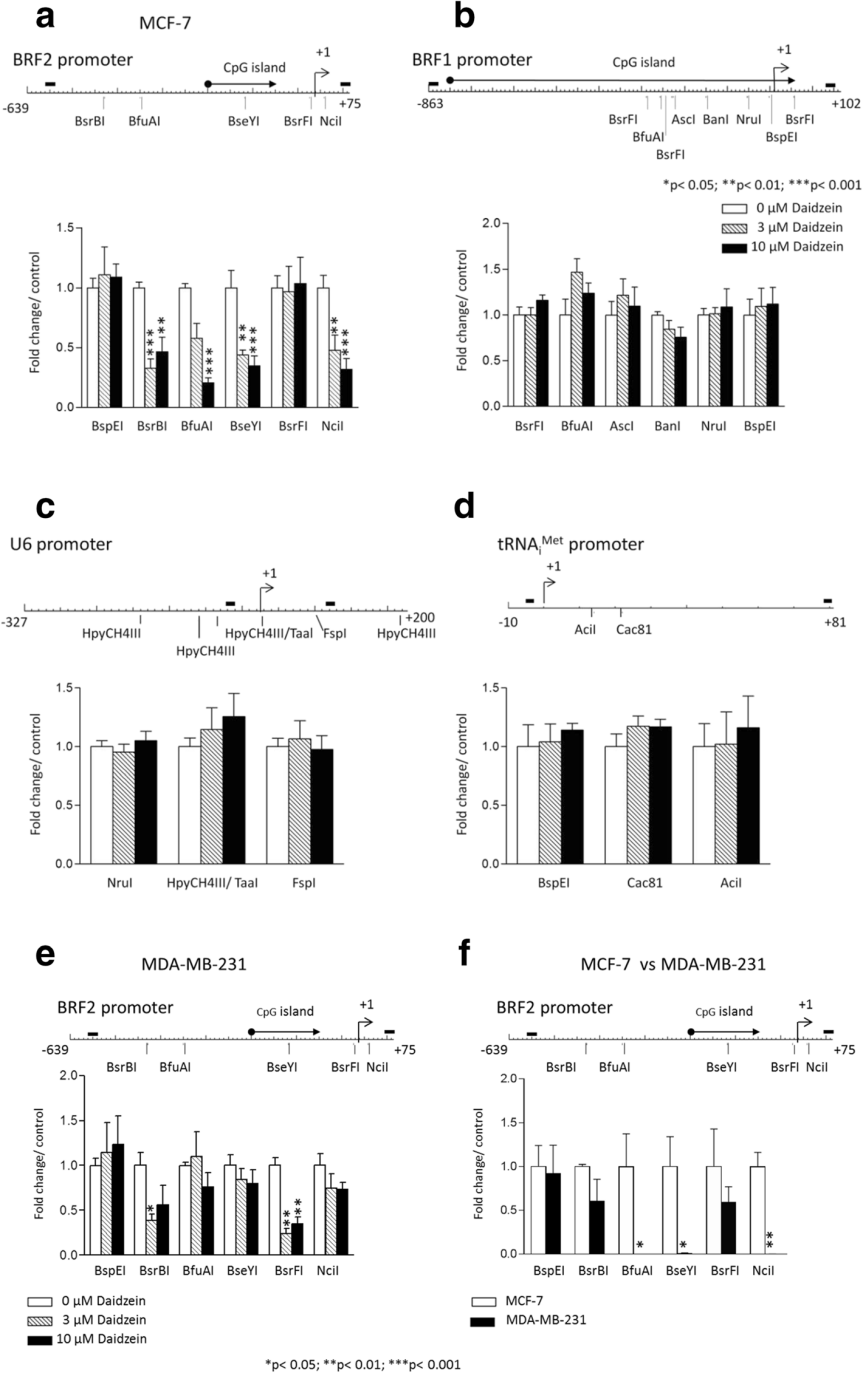
Daidzein changes DNA methylation at the BRF2 promoter specifically. CpG methylation- sensitive restriction enzymes cut sites are shown. Transcription start site (TSS) is indicated by +1. MethPrimer program was used to predict the location of CpG islands within the BRF2 (402–556 bp) and BRF1 (50–910 bp) promoters, as indicated by black arrow. Black bars denote the binding sites for primers used in the methylation profile analysis. **a-d** MCF-7 and (**e**) MDA-MB-231 cells were treated with 0, 3, 10 μM daidzein for 48 h. The genomic DNA was harvested and digested with methylation-sensitive restriction enzymes with cut sites within the promoter, noted on BRF2 and BRF1 promoter schematic, and as a negative control one methylation sensitive enzyme with no recognition sites in the promoter was used. BspEI and BsRFI do not have recognition sites in BRF2 and BRF1 promoters, respectively. The digestion profile was then analyzed by qPCR using primers spanning the BRF2 (**a**, **e, f**) and BRF1 (**b**) promoter regions, U6 (**c**) and tRNA_i_^Met^ (**d**) genes. DNA methylation levels were calculated using ΔΔCt method with RPS13 expression levels used as a reference for normalization. Data presented are average of three independent experiments. Statistical analysis was performed using one-way ANOVA a Tukey’s post-test with a 95 % confidence interval (Graphpad Prism 3.03); * = *p* <0.05; ** = *p* < 0.01; *** = *p* < 0.001

There are a number of published studies describing epigenetic regulation of gene expression by dietary polyphenols in prostate and breast cancer cell lines [44, 45]. We therefore investigated whether daidzein influences DNA methylation of the BRF1 and BRF2 promoter regions. Treatment of MCF-7 cells with 10 μM daidzein significantly decreased methylation of four restriction sites in the BRF2 promoter (Fig. 4a). The same treatment had minimal effect on the methylation status of several restriction sites within the BRF1 promoter (Fig. 4b). The minimal response of BRF1 indicates that the effect of daidzein on BRF2 promoter methylation is selective. Further evidence of specificity is provided by analysis of U6 and tRNA_i_^Met^ genes, where DNA methylation is unchanged in response to daidzein (Figs. 4c and d). Localized demethylation of the BRF2 promoter was also observed when MDA-MB-231 cells were treated with daidzein, but the response was less marked than in MCF-7 cells and only one site showed a statistically-significant change at the 10 μM dose (Fig. 4e). In untreated cells, the BRF2 was much less heavily methylated in MDA-MB-231 than in MCF-7 (Fig. 4f), which may explain its higher expression in the former (Fig 2. and [42]).

**Fig. 5:**
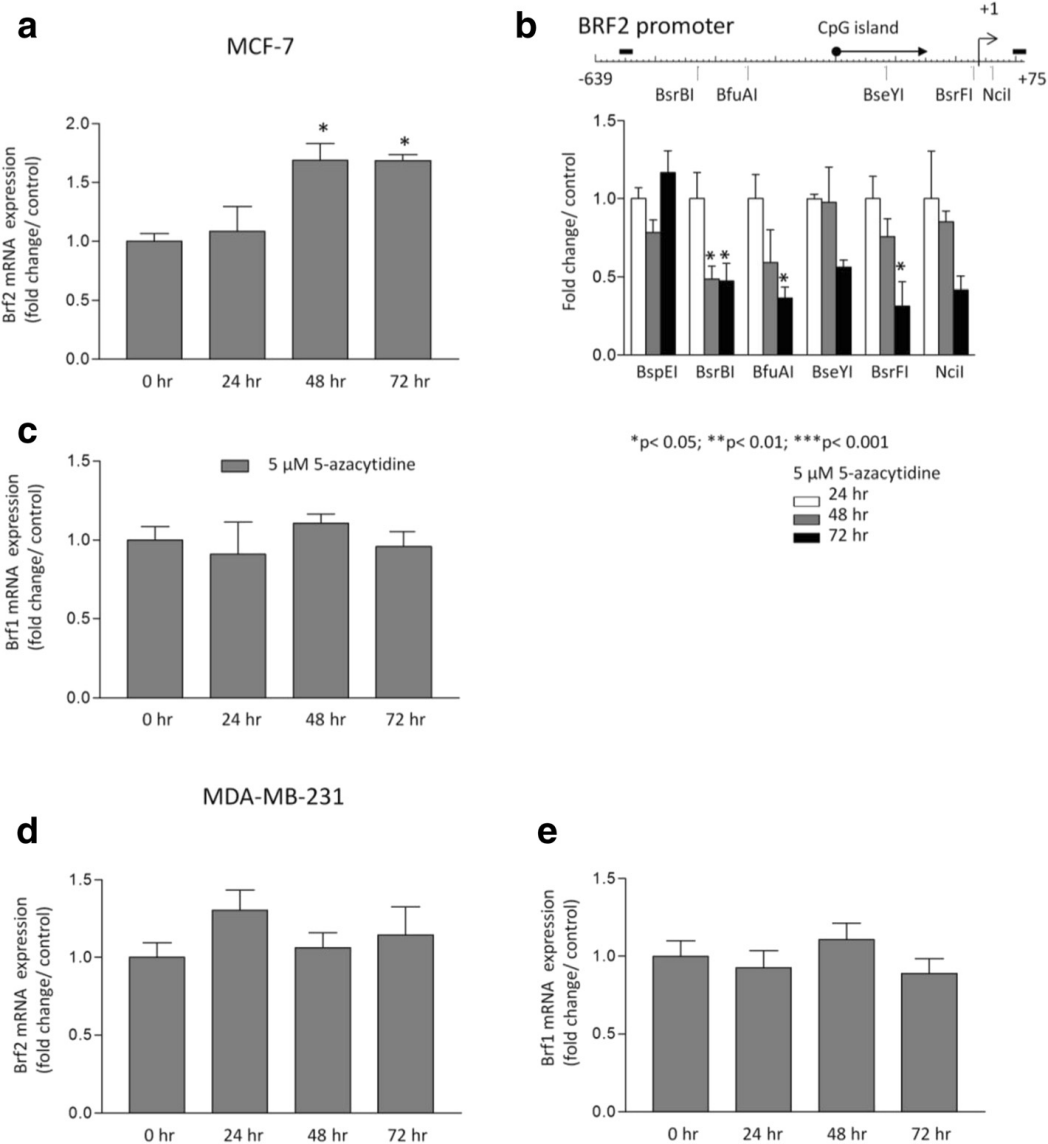
Azacytidine induces demethylation and expression of BRF2 selectively in MCF-7 cells. **a, b, c** MCF-7 and (**d, e**) MDA-MB-231 cells were treated with 5 μM 5-Azacytidine for 24, 48 and 72 h. Post-treatments, total RNA was isolated and analyzed by qRT-PCRfor expression of (**a, d**) BRF2 and (**c, e**) BRF1 mRNAs. The ΔΔCt method with GAPDH and RPS13 expression levels was used as a reference for normalization. Meta-analysis of two independent experiments performed in triplicate was completed using one-way ANOVA with a Tukey’s post-test with a 95 % confidence interval (Graphpad Prism 3.03); * = *p* <0.05; ** = *p* < 0.01; *** = *p* < 0.001. (**b**) Genomic methylation of BRF2 promoter DNA in MCF-7 cells was analyzed with methylation-sensitive restriction enzymes, as in Fig. 4a

If demethylation of promoter DNA can influence transcription of the BRF2 gene, then treatment with the methylation inhibitor 5-azacytidine might be predicted to induce expression. Indeed, BRF2 mRNA levels were elevated within 48 h of adding 5-azacytidine to MCF-7 cells, along with promoter demethylation (Figs. 5a and b). This response was specific, as it was not shown by BRF1 mRNA when assayed in parallel (Fig. 5c). Furthermore, the same treatment failed to induce either BRF1 or BRF2 in MDA-MB-231 cells (Figs. 5d and e). These data suggest that BRF2 promoter activity may be sensitive to DNA methylation, which responds to daidzein in MCF-7 cells.

### Daidzein increases stability of BRF1 and BRF2 mRNAs in MCF-7 cells

**Fig. 6:**
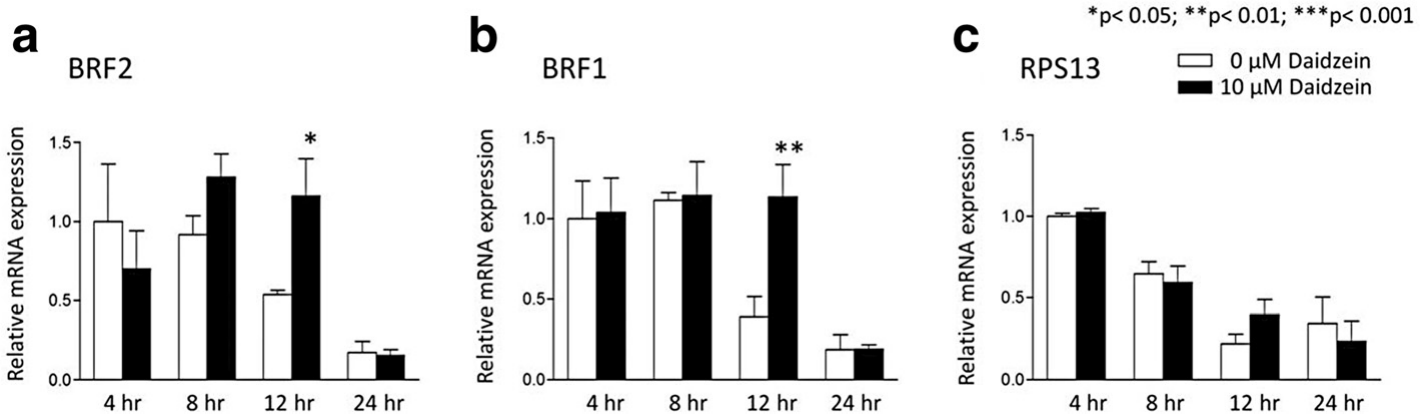
Daidzein increases stability of BRF1 and BRF2 mRNAs selectively in MCF-7 cells. MCF-7 cells were treated with 5 μg/ml of actinomycin D, either alone (white bars) or with 10 μM daidzein (black bars) for 24 h. The cells were then harvested at 4, 8, 12 and 24 h. The RNA was extracted and analyzed by qRT-PCRfor levels of (**a**) BRF2, (**b**) BRF1, (**c**) RPS13, and actin β mRNAs. ΔΔCt method with RPS13 and actin β expression levels were calculated for assay normalization. Data presented are average of three independent experiments. Statistical analysis was performed using one-way ANOVA with a Tukey’s post-test with a 95 % confidence interval (Graphpad Prism 3.03); * = *p* <0.05; ** = *p* < 0.01; *** = *p* < 0.001

The 3’-untranslated regions (3’-UTR) of BRF1 and BRF2 mRNAs are AU-rich, which may influence their stability [42]. Various classes of polyphenols have been shown to post-transcriptionally regulate levels of mRNA containing AU-rich elements by raising expression of RNA binding proteins [46–49]. To test if daidzein stabilizes BRF mRNAs, as a potential mechanism of induction, we treated MCF-7 cells with the transcription inhibitor actinomycin D [41] and monitored mRNA decay using qRT-PCR. Although 10 μM daidzein was unable to prevent the turnover of BRF mRNAs by 24 h, it prevented degradation of both BRF2 (p < 0.01; Fig. 6a) and BRF1 (*p* < 0.05; Fig. 6b) for 12 h after transcription inhibition. This effect is specific, as mRNA encoding ribosomal protein S13 (RPS13) decayed with similar kinetics in the presence or absence of daidzein, when assayed in parallel (Fig. 6c). The selective stabilization of BRF mRNAs offers a potential explanation for the increased expression seen when MCF-7 cells are exposed to 10 μM daidzein. Additional mechanisms may also be involved, such as demethylation of the BRF2 promoter (Fig. 4b).

### Commercial rodent chow differentially regulates BRF1 and BRF2 in a sex-dependent manner

**Fig. 7:**
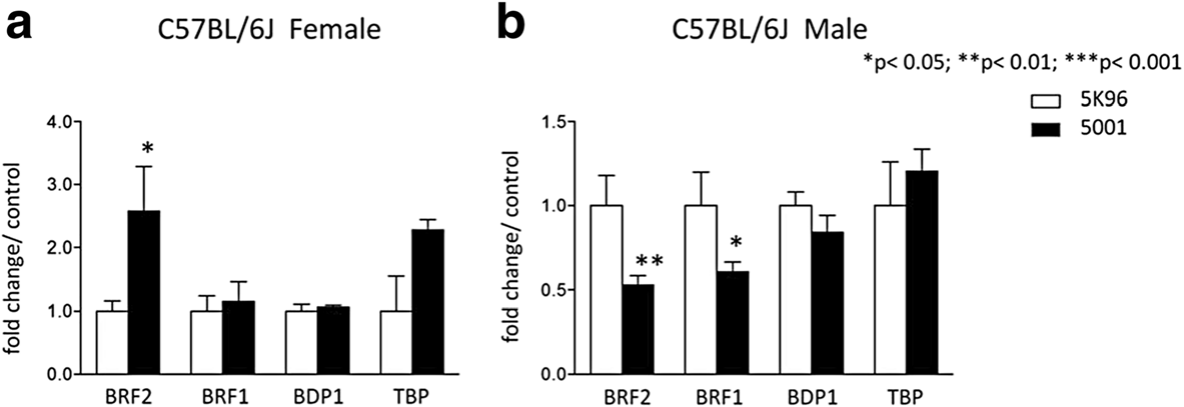
Differential expression of TFIIIB subunits in female and male mice fed high or low isoflavone diets. Five week C57CBL/6 J (**a**) female and (**b**) male mice had free access to isoflavone-free, casein-based diet 5 K96 or regular Lab Diet 5001, with high isoflavone content. Post-treatments, total RNA was isolated from blood collected by cardiac puncture and analyzed by qRT-PCR for expression of BRF2, BRF1, BDP1, and TBP. The ΔΔCt method with GAPDH expression levels was used as a reference for normalization. Data presented are average of six female and six male mice from respective groups. Statistical analysis was performed using one-way ANOVA with a Tukey’s post-test with a 95 % confidence interval (Graphpad Prism 3.03); * = p <0.05; ** = p < 0.01; *** = p < 0.001

Purina 5001 is a chow that is commonly fed to laboratory rodents and contains high levels of both daidzein and genistein, the two major isoflavone components of soybeans [50]. Our observations with daidzein in cultured cells prompted us to investigate if BRF expression is elevated in mice fed a diet enriched in these isoflavones. RNA was isolated from the blood of mice maintained on either Purine 5001 or the isoflavone-free casein-based Purina 5 K96 chow. Significantly elevated BRF2 expression was found in female mice with the high isoflavone Purina 5001 diet (Fig. 7a). In striking contrast, the opposite was seen in males, where BRF2 expression was significantly lower with high isoflavones relative to the isoflavone-free diet (Fig. 7b). BRF1 was also suppressed in males fed the high isoflavone Purina 5001 chow, but was unaffected in females. No statistically significant changes were found in the other TFIIIB subunits, TBP and BDP1. These *in vivo* data suggest that male and female C57BL/6 J mice respond differently to dietary sources of isoflavones and these differences are measurable in the blood. They emphasize the importance of taking into account both gender and diet in mouse models of cancer.

## Discussion

Breast cancer is the leading cause of cancer-related deaths in females in developing countries and the second leading cause in developed countries [51, 52]. It has been estimated that there will be approximately 1.7 million new cases of breast cancer in 2020, a 26 % increase from current incidence rates, mostly due to the increase in new cases in the developing world [53]. As diet is known to influence the incidence of breast cancer [54], identifying the impact of specific foods is imperative. Soy has been studied extensively for its anti-cancer properties, but the epidemiological results have been contradictory, in part because soy can regulate gene expression via estrogen- dependent and independent pathways. We therefore investigated the molecular effects of the isoflavone daidzein in both ER-positive and ER-negative cancer cells. We observed clear effects in the former that were not seen in the latter. Specifically, daidzein raises expression of BRF2 and BRF1 in MCF-7 cells, as well as pol III transcripts that depend on these TFIIIB subunits for their synthesis. It is noteworthy that another polyphenol, EGCG that is enriched in green tea, also affects expression of BRF1 and BRF2 selectively, but in this case elicits an inhibitory effect, in keeping with its anti-cancer activity [31].

Induction of BRF2 and BRF1 by daidzein may be explained by stabilization of their respective mRNAs, although additional effects might also contribute. For example, we also observed demethylation of the BRF2 promoter that might result in a transcriptional response. How the polyphenol produces these effects remains to be established. A clear possibility is that the ER is involved, either directly or indirectly, as this can be weakly bound by daidzein [33]. Indeed, ER has been shown to bind directly to the BRF1 promoter in MCF-7 cells [55] and 17β-estradiol has been shown to induce tRNA synthesis [56]. Those studies did not investigate BRF2 or its target genes, such as U6. Circumstantial evidence in support of ER involvement is provided by the differential response in ER-positive MCF-7 and ER-negative MDA-MB-231 cells in culture, and the gender-specificity of induction in mice. However, further studies will be required to establish if ER mediates the observed effects. In terms of the current study, we consider the crucial finding to be that physiologically relevant doses of a common dietary compound can stimulate expression of a gene with established oncogenic properties. This raises concerns for women consuming soy.

It remains to be determined how BRF2 exerts oncogenic effects, although the obvious possibility is through transcriptomic changes, given its well-established function as a transcription factor. Genome-wide analyses showed that BRF2 has a highly restricted set of target genes [57], all encoding short pol III-dependent noncoding RNAs. In addition to U6 snRNA, these include 7SK RNA, which regulates transcription of protein-coding genes by inhibiting P-TEFb [58–60], and hY RNAs, which promote DNA replication [61]. The hY RNAs are commonly overexpressed in tumors [62, 63]. For example, hY1 is overexpressed ~13-fold in lung, prostate and other cancers (*P* = 9.7 × 10^−26^) and its knockdown inhibits proliferation of lung and prostate cell lines [62]. As a key component of spliceosomes, U6 snRNA is an essential BRF2-dependent product. It was consistently detected at elevated levels in sera from 140 breast cancer patients, relative to 115 healthy age-matched controls [64]. U6 was also the most strongly upregulated (P < 0.0001; FDR <0.001) non-coding RNA in serum from 75 glioblastoma multiforme patients, relative to matched healthy controls [65]. MicroRNAs are widely believed to have considerable potential as biomarkers, but U6 outperformed all 381 miRNAs tested in this study [65]. It has also been suggested that BRF2 might serve as a biomarker, given its early induction in development of lung SqCC [22], where high BRF2 is independently prognostic (*P* = 0.007) of unfavourable survival [24], as also reported (*P* = 0.009) for oesophageal SqCC [23]. Our demonstration that BRF2 levels can be monitored in mouse serum provides initial evidence that a non-invasive test is practical for this potential biomarker.

## Conclusions

This study demonstrates, both *in vitro* and *in vivo*, that a common soy isoflavone, at dietary concentrations, can influence expression of BRF2 and BRF1, essential components of the pol III-specific transcription factor TFIIIB. The response may involve an epigenetic component and gender-dependence in mice. Its importance is suggested by recent evidence that BRF2 can be oncogenic and prognostic of poor survival.

### Ethics

Animal experiments presented were approved by SJU IACUC protocol 1831.0 in its entirety.

### Availability of supporting data

Authors declare there is no additional supporting data to be made available. All DNA promoter sequences are readily available in PubMed database.
